# Data-driven gating (DDG)-based motion match for improved CTAC registration

**DOI:** 10.1186/s40658-024-00644-0

**Published:** 2024-05-01

**Authors:** Ella L. Cook, Kuan-Hao Su, Geoff S. Higgins, Robert Johnsen, Jean-Paul Bouhnik, Daniel R. McGowan

**Affiliations:** 1 Department of Oncology, University of Oxford, Oxford, UK; 2 GE HealthCare, Waukesha, Wisconsin USA; 3 GE HealthCare, Haifa, Israel; 4 Department of Medical Physics and Clinical Engineering, Oxford University Hospitals Foundation Trust, Oxford, UK

**Keywords:** Respiratory motion, Data-driven gating, 4DCT

## Abstract

**Background:**

Respiratory motion artefacts are a pitfall in thoracic PET/CT imaging. A source of these motion artefacts within PET images is the CT used for attenuation correction of the images. The arbitrary respiratory phase in which the helical CT ($\hbox{CT}_{\text{helical}}$) is acquired often causes misregistration between PET and CT images, leading to inaccurate attenuation correction of the PET image. As a result, errors in tumour delineation or lesion uptake values can occur. To minimise the effect of motion in PET/CT imaging, a data-driven gating (DDG)-based motion match (MM) algorithm has been developed that estimates the phase of the $\hbox{CT}_{\text{helical}}$, and subsequently warps this CT to a given phase of the respiratory cycle, allowing it to be phase-matched to the PET. A set of data was used which had four-dimensional CT (4DCT) acquired alongside PET/CT. The 4DCT allowed ground truth CT phases to be generated and compared to the algorithm-generated motion match CT (MMCT). Measurements of liver and lesion margin positions were taken across CT images to determine any differences and establish how well the algorithm performed concerning warping the $\hbox{CT}_{\text{helical}}$ to a given phase (end-of-expiration, EE).

**Results:**

Whilst there was a minor significance in the liver measurement between the 4DCT and MMCT ($p = 0.045$), no significant differences were found between the 4DCT or MMCT for lesion measurements ($p = 1.0$). In all instances, the $\hbox{CT}_{\text{helical}}$ was found to be significantly different from the 4DCT ($p < 0.001$). Consequently, the 4DCT and MMCT can be considered equivalent with respect to warped CT generation, showing the DDG-based MM algorithm to be successful.

**Conclusion:**

The MM algorithm successfully enables the phase-matching of a $\hbox{CT}_{\text{helical}}$ to the EE of a ground truth 4DCT. This would reduce the motion artefacts caused by PET/CT registration without requiring additional patient dose (required for a 4DCT).

## Background

CT attenuation correction (CTAC) is the most prominent correction applied to PET data within PET/CT [1]. The use of the CT creates an attenuation map of the various tissue densities and associated photon absorption allowing for accurate lesion quantification and monitoring through the use of features such as the standardised uptake value (SUV). However, there is scope for error due to the high dependence on the CT, as it must be accurately aligned to the PET acquisition. Any misalignments of the CT, such as those due to gross or respiratory patient motion, will detriment the PET image, through misregistration between the continuously acquired PET and temporally discrete helical CT ($\hbox{CT}_{\text{helical}}$) acquisition at an arbitrary phase. The potential CTAC misregistration will affect lesion quantification and diagnostic accuracy [2, 3].

Previous research into motion correction techniques for PET/CT include the assessment of external device-based gating methods with software-based approaches such as data-driven gating (DDG) and has been reviewed elsewhere [4–7]. Additional techniques concerning the improvement of image quality of those subject to motion have been studied through various reconstruction algorithms and commercialised products have been developed as a result [4, 8–12]. However, previous literature does not conclude a gold standard motion correction technique used across the field to date, with many being manufacturer-specific. Most methods address modifications to the PET acquisition and reconstruction where considerations of the CT impact on the quantified PET image are often overlooked [2]. Our study aims to investigate improvements to the CT phase misalignment from PET, capitalising on a dataset containing four-dimensional CT (4DCT) data as a ground-truth comparison to the novel approach.

The difference in scan acquisition duration of each imaging modality encourages the misalignment and subsequent misregistration of the PET and CT images due to inter- and intra-scan motion negatively affecting quantitative output [13]. Motion examples can derive from patient discomfort, or claustrophobia, giving rise to image misalignment within the PET/CT images. In addition, the PET acquisition time of several minutes takes place over many respiratory cycles compared to the CT, which can take place over a sub-second range. Therefore, for regions within the thorax and abdomen, respiratory motion is likely a significant factor in the impact on image quality [4, 14–16]. This reduction in image quality typically occurs as a blurring of the emission data and the reduction of the SUV and increased lesion volume [4].

The R-value is a metric for each bed position, determining the respiratory-like motion within it, and is a quantitative threshold for suitable clinical application of motion correction techniques [8]. R-values are calculated in Fourier domain using respiratory waveforms where the value is the ratio of the peak value within the respiratory frequencies (0.1–0.4 Hz) from each principal component with the mean value beyond this frequency range. This ratio is indicative of respiratory motion within the PET list data [9, 17]. R = 15 is the widely accepted threshold value for a manufacturer’s respiratory gating algorithm (MotionFree, GE HealthCare) [4, 11, 17].

A 4DCT acquisition occurs over a longer duration, rather than taking an image at a stationary point in time, and requires a higher patient dose, approximately five times greater than a $\hbox{CT}_{\text{helical}}$. Phase-matching allows the alignment of the correct PET phase from a particular time with the corresponding CT phase (derived from 4DCT), providing a more accurate measure of attenuation correction [18]. Given recent advancements in 4DCT application, motion correction has become more successful, leading to more accurate lesion quantification through respiratory-correlated CT use [18–20]. Such improved attenuation correction accuracy results in improved diagnostic accuracy. The major drawback of the 4DCT is the additional patient dose compared to $\hbox{CT}_{\text{helical}}$ and the additional patient set up generally required.

Existing methods have been considered for PET/CT motion correction and misregistration improvements with evolution from external device-based to deviceless technologies i.e. DDG. Many current phase-matching methods still depend on 4DCT, either external device- or DDG-based, acquiring a cine CT over a respiratory cycle and increasing patient dose. Conventional phase-matching methods utilise external devices to extract respiratory signals from the cine CT and deviceless methods use metrics from Hounsfield Units (HU) of lung CT density but still require cine CT acquisition [11, 12, 21]. The proposed motion match (MM) algorithm warps a widely available, free-breathing $\hbox{CT}_{\text{helical}}$ to generate a motion-matched CT (MMCT), representing the phase best matching the PET data, reducing misregistration. MMCT does not require a 4DCT acquisition over a respiratory cycle as with 4DCT CTAC. The MMCT algorithm objective is to demonstrate a deviceless technique to improve CTAC registration without excess patient exposures.

## Methods

Patient data was acquired from research imaging studies investigating tumour hypoxia in non-small cell lung cancer (NSCLC) of adult patients using 4D $^{18}\hbox{F}$-fluoromisonidazole ($^{18}\hbox{F}$-FMISO) PET/CT (e.g. ATOM, [22]) at the Churchill Hospital Oxford. Patients were injected with an activity of 370 MBq ± 10% of $^{18}\hbox{F}$-FMISO (University of Cambridge, Cambridge, England, United Kingdom) prior to imaging. 4DCT images (six gates) were obtained in the studies to calculate the extent of respiratory motion.

In this study, patients are deemed evaluable via the R-value, used to determine whether to use PET respiratory gating [11]. Patients $\text{R}>12$ were included in this study ($\text{n}=18$), details in Table 1.

**Table 1:** Table detailing R-value distributions throughout the patient cohort

	R-value	n
	$12>R>14.99$	9
	$15>R>19.99$	7
	$R\ge 20$	2
Total	$12>R>40$	18

A lower R threshold was used (R > 15 is typically used clinically) to maximise the available dataset. The R-value of patients included varied from 12.1 to 25.8 with a mean value, $\bar{R} = 15.8$.

### Image acquisition and reconstructions

The image datasets were acquired using a Discovery D710 PET scanner (GE HealthCare). The field-of-view (FOV) comprised a single bed position from the superior thorax to the superior abdomen, imaging the lung, heart and liver. Patients were scanned using 10-minute PET acquisitions, two and four hours following $^{18}\hbox{F}$-FMISO injection [22]. The PET data in this study was corrected for motion using the manufacturer’s DDG-based MotionFree [4, 11].

The 4DCT images were generated using an external Real-time Position $\hbox{Management}^{\text{TM}}$ (RPM) device, secured on the patient’s abdomen. The 4DCTs were conducted under free-breathing conditions where patients were advised to maintain regular breathing, if possible. The static $\hbox{CT}_{\text{helical}}$ is automatically initiated 30 s after 4DCT acquisition, also under free-breathing conditions. The 4DCT data was acquired as a series of 4 cm axial-width cine CTs (across the PET axial FOV) then binned into six gates using MotionMatch (GE HealthCare) without any user-guided improvement from default to determine the gates. A single series over six phases or gates were saved, encompassing the entire regular patient respiratory cycle from end-of-inspiration (EI) of one cycle to EI of the next cycle, across the six gates. This binning ensured that the investigated EE phase was entirely acquired.

### Motion match algorithm

The warped motion-matched PET/CT reconstructions were performed using an offline reconstruction package, Duetto (GE HealthCare toolbox in MATLAB), which is part of a research agreement and not an available product. To create the warped CT, the MM algorithm must first approximate the initial phase within the respiratory cycle that $\hbox{CT}_{\text{helical}}$ was acquired.

**Fig. 1:**
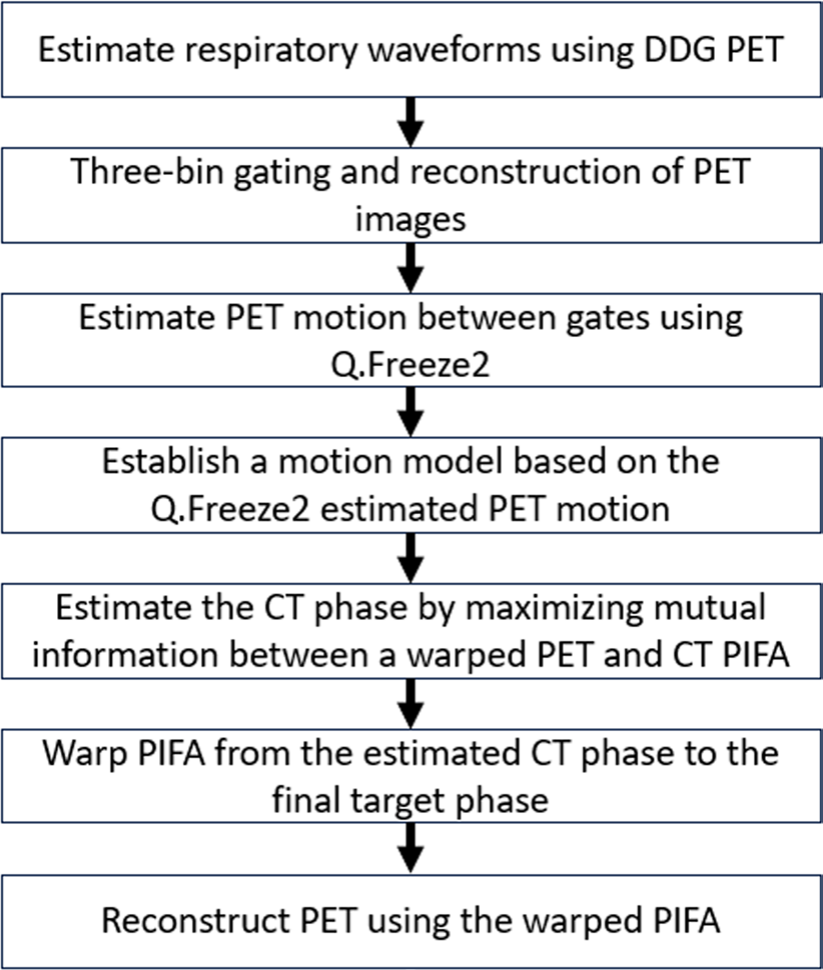
Flowchart outlining MMCT algorithm workflow

The MM algorithm uses an adaptation of the manufacturer’s Bayesian penalized likelihood (PL) reconstruction algorithm ($\beta = 350$), Q.Clear, a non-ToF BSREM algorithm without attenuation correction information for motion estimation (number of reconstruction voxels in the x–y dimension, $\hbox{nX} = 64$, $\hbox{FOV} = 70$ cm) [23]. A ToF BSREM algorithm was used to reconsruct the final MM corrected images. The algorithm’s workflow is indicated in Fig. 1.

- The three-bin gated PET images and respiratory waveforms were extracted directly from the listmode and raw data files using principal component analysis (PCA) allowing for respiratory motion model generation.
- Three bins are selected for comprehensive coverage of each phase of the respiratory cycle. An essential difference between the 4DCT and MMCT is the binning method; the 4DCT uses phase-based binning, whereas MMCT uses amplitude-based binning. Phase-based binning assigns the breathing cycle over $2\pi$ radians [24]. Amplitude-based gating is based upon the division of the respiratory signal with respect to the magnitude of the patients’ respiratory motion [25]. EE and EI of each respiratory cycle are defined by amplitude-based gating scales as 0% amplitude and 100% amplitude, respectively. Amplitude-based gating of PET was used over phase-based gating due to improved determination of the quiescent phase in patients with irregular breathing patterns by using respiratory triggers and waveform geometry [26, 27].
- The deformation fields across different gates of the PET data were estimated using the manufacturer’s Q.Freeze2 algorithm [12].
- The motion model was established by assuming a linear relationship between respiratory phases and deformation amongst gates. This model allows generation of PET images at any target phase, where 28 PET images at equally sampled phases between 0 and 200% were generated. The phase range was extended to 200% to overcome the fact that patients can sometimes inhale deeper but rarely exhale deeper in CT acquisition compared to PET.
- Using the deformation field amongst gates to warp PET images into any arbitrary phases enables the estimation of a CT phase by maximising mutual information between the warped PET and PET Image For Attenuation (PIFA) data, where PIFAs are 511 keV attenuation coefficient images converted from CT.
- Following CT phase estimation, new warped PIFAs and subsequent CT DICOMs can be generated for any desired target phase of the respiratory cycle based on the motion model estimated from PET.
- These warped images can then be used for the CTAC in PET reconstructions, reducing the extent of misregistration in PET/CT with scope for improvements in PET quantification.

### Analysis of CT images

**Fig. 2:**
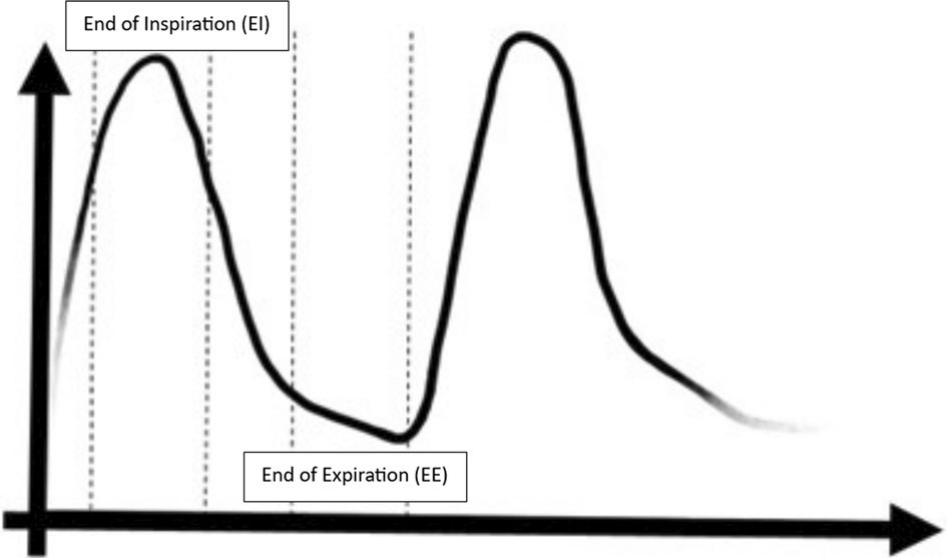
Schematic illustrating typical respiratory waveform, with EI and EE segments for the first waveform marked

The end-of-expiration (EE) phase is subject to minimal motion compared to other parts of the respiratory cycle, as seen in Fig. 2.

In order to determine the differences between the CTs used for CTAC, multiple methods could be implemented to assess the respective differences. In this work, the EE phase of the 4DCT will be treated as the ground truth and compared to $\hbox{CT}_{\text{helical}}$ (an image acquired within an arbitrary phase of the respiratory cycle) and the EE phase of morphed MMCT (derived from $\hbox{CT}_{\text{helical}}$ using Duetto). Due to the differences in amplitude and phase-based gating, the EE phase is consistent between each gating method. In addition, it is the phase generally used when correcting for respiratory motion in reconstructing a quiescent phase PET/CT image.

Reconstructed CT images were analysed in Affinity (Hermes Medical Solutions) and measurements of different anatomical markers were taken to determine the differences between the CTACs. These measurements were taken for each exam of the liver and lesions, as these are key indicators of motion throughout the patient’s respiratory cycle. The relevant anatomical measurements to determine displacement are:

- Superior liver margin to bottom image boundary
- Superior lesion margin to top image boundary
- Inferior lesion margin to top image boundary

**Fig. 3:**
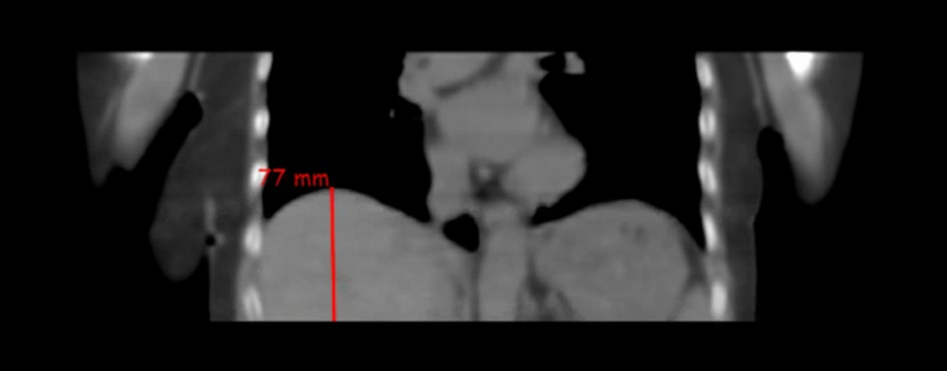
CT image illustrating displacement measurement of the superior liver to inferior image boundary, representing measurement (**a**)

An example of these measurements is given in Fig. 3.

### Statistical analysis

The raw data collected has a non-normal distribution due to inter-variability between patient’s unique disease burden and anatomy. To improve consistency across all patients, data for the various investigative parameters was collected as a percentage change relative to the result acquired for a particular group, i.e. 4DCT or $\hbox{CT}_{\text{helical}}$ for CTAC comparisons. This results in more normally distributed data. These corrections improve accuracy and validity in the statistical tests used during analysis, where parametric tests are used. Data normality is assessed by using the Shapiro-Wilk test, best suited due to the small sample size of respective datasets.

Given the normal data distribution upon transformation into percentage change, the parametric, repeated-measures, one-way, ANOVA is used to test for differences within the dataset due to comparison of three or more groups.

From the given results, the null hypothesis can be rejected for significance values of $\it p \le 0.05$ and accepted for values $\it p > 0.05$. For cases with a sphericity violation, as per the Mauchly test, a Greenhouse-Geisser correction was considered before concluding any significant results. Where results were deemed significant, post hoc analysis was completed using paired *t*-tests between groups.

For comparison of multiple groups, significance levels are adjusted using Bonferroni corrections, so any value $p \le 0.05$ can be deemed statistically significant. Analysis was conducted using SPSS v29.

## Results

The CTs’ initial phase estimation is an important consideration for changes observed between CTACs. This dataset has a mean value of 70% with a range of 0–122% on the amplitude-based gating scale. Suppose a $\hbox{CT}_{\text{helical}}$ was already acquired during EE; there will likely be less significant differences in CTAC and associated effects than if $\hbox{CT}_{\text{helical}}$ was acquired at other cycle phases. Consequently, more significant changes are likely to be seen at the EI phase, or greater than 100% on the amplitude-based gating scale.

**Fig. 4:**
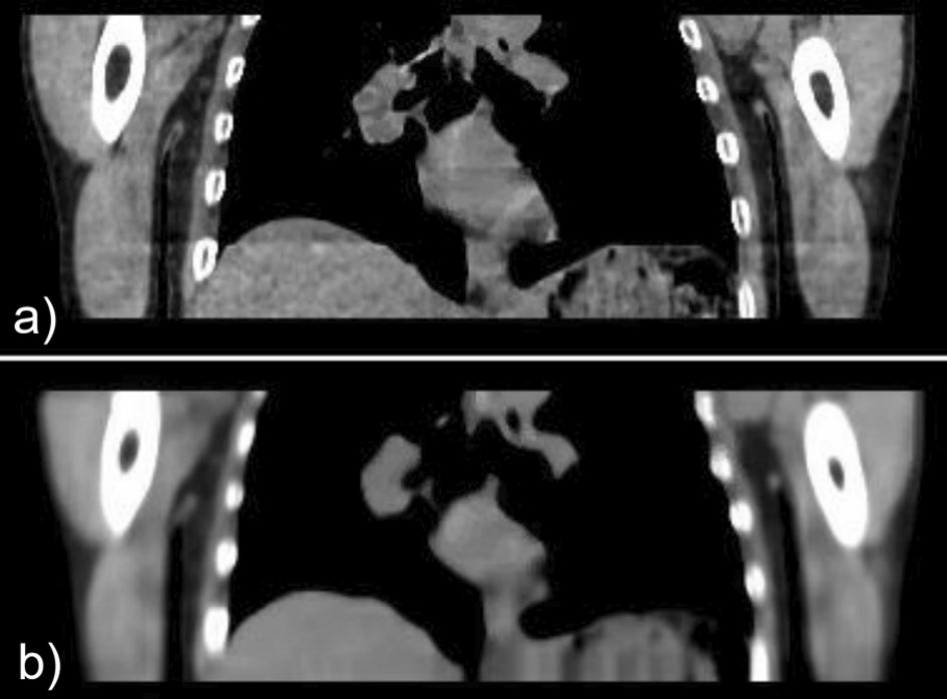
4DCT (**a**) and MMCT (**b**) for an example patient

An example MMCT and 4DCT shown in Fig. 4, with Table 2 summarising pairwise comparisons of percentage change in positional measurements between CTAC groups.

**Table 2:** Pairwise comparisons of 4DCT against $\hbox{CT}_{\text{helical}}$ and MMCT conditions, acquired using a repeated-measures, one-way, ANOVA

Pairwise comparison (parameter/4DCT with...)	Mean difference (4DCT-Other)	25th/75th Percentile	Significance ($p = \ldots$)
Superior liver margin/$\hbox{CT}_{\text{helical}}$	14.8 ± 3.1	6.5/23.2	< 0.001
Superior liver margin/MMCT	6.1 ± 2.2	0.1/12.2	0.045
Superior lesion margin/$\hbox{CT}_{\text{helical}}$	− 11.2 ± 3.1	$-19.6$/$-2.9$	0.007
Superior lesion margin/MMCT	0.3 ± 1.4	$-3.5$/4.1	1.0
Inferior lesion margin/$\hbox{CT}_{\text{helical}}$	− 4.9 ± 0.7	$-6.7$/$-3.1$	< 0.001
Inferior lesion margin/MMCT	0.2 ± 0.6	$-1.5$/2.0	1.0

Parameters are expressed as a percentage change

### Superior liver margin position

**Fig. 5:**
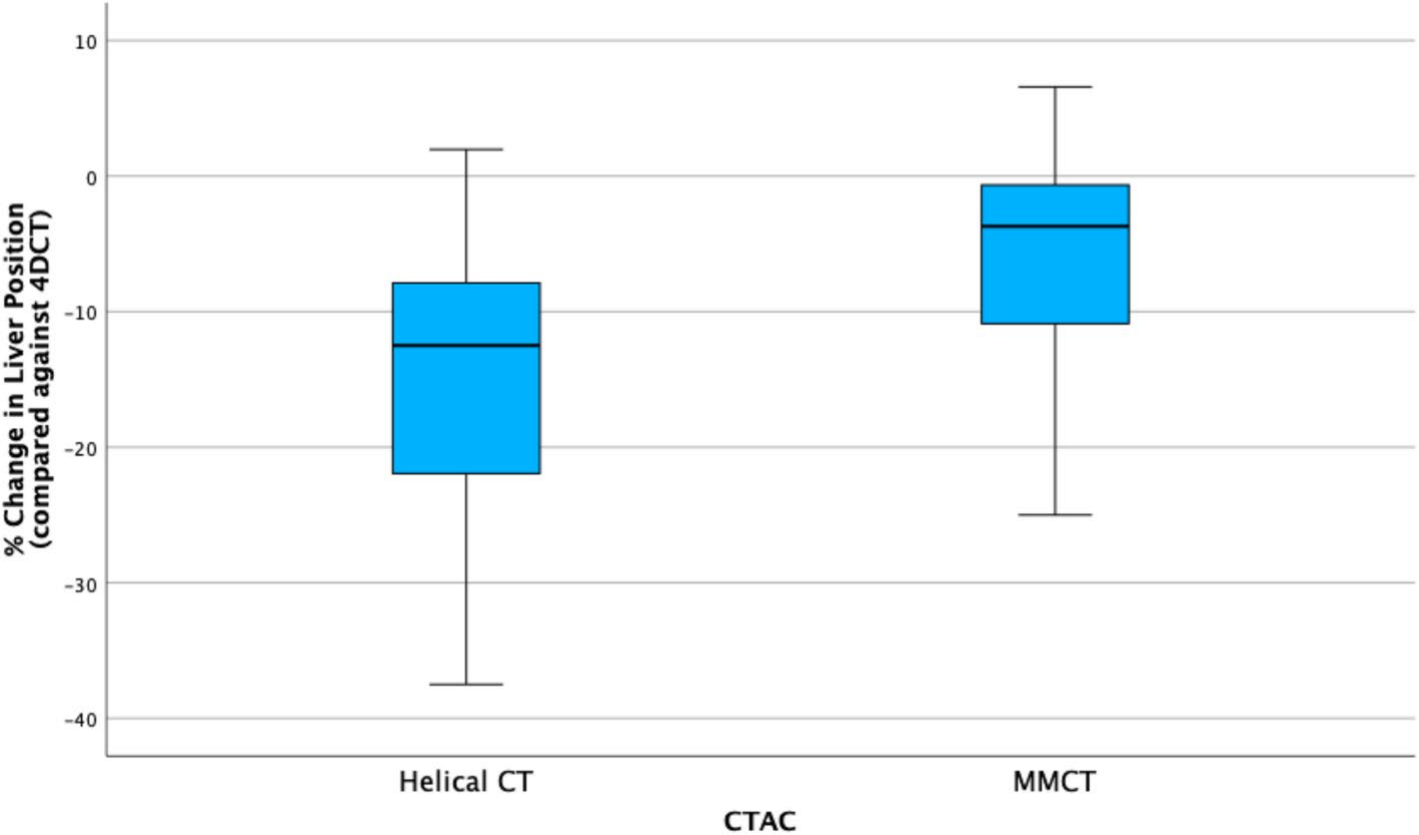
Boxplot illustrating differences in percentage change in superior liver margin for $\hbox{CT}_{\text{helical}}$ and MMCT CTAC when compared against the 4DCT group for EE

The repeated-measures one-way ANOVA indicates a statistically significant difference in liver position within CTAC groups (Wilks’ $\lambda = 0.037$, $\hbox{F}(1.42, 19.83) = 18.90$, $p < 0.001$), illustrated in Fig. 5.

Greenhouse-Geisser corrections have been applied due to sphericity violation within the dataset. Further analysis using post hoc tests denotes there are statistically significant differences between 4DCT group and $\hbox{CT}_{\text{helical}}$ group ($p < 0.001$) and 4DCT group and MMCT group ($p = 0.045$). The mean difference within MMCT group is less than $\hbox{CT}_{\text{helical}}$ group, which suggests MMCT provides a closer approximation to 4DCT than $\hbox{CT}_{\text{helical}}$.

### Superior lesion margin position

**Fig. 6:**
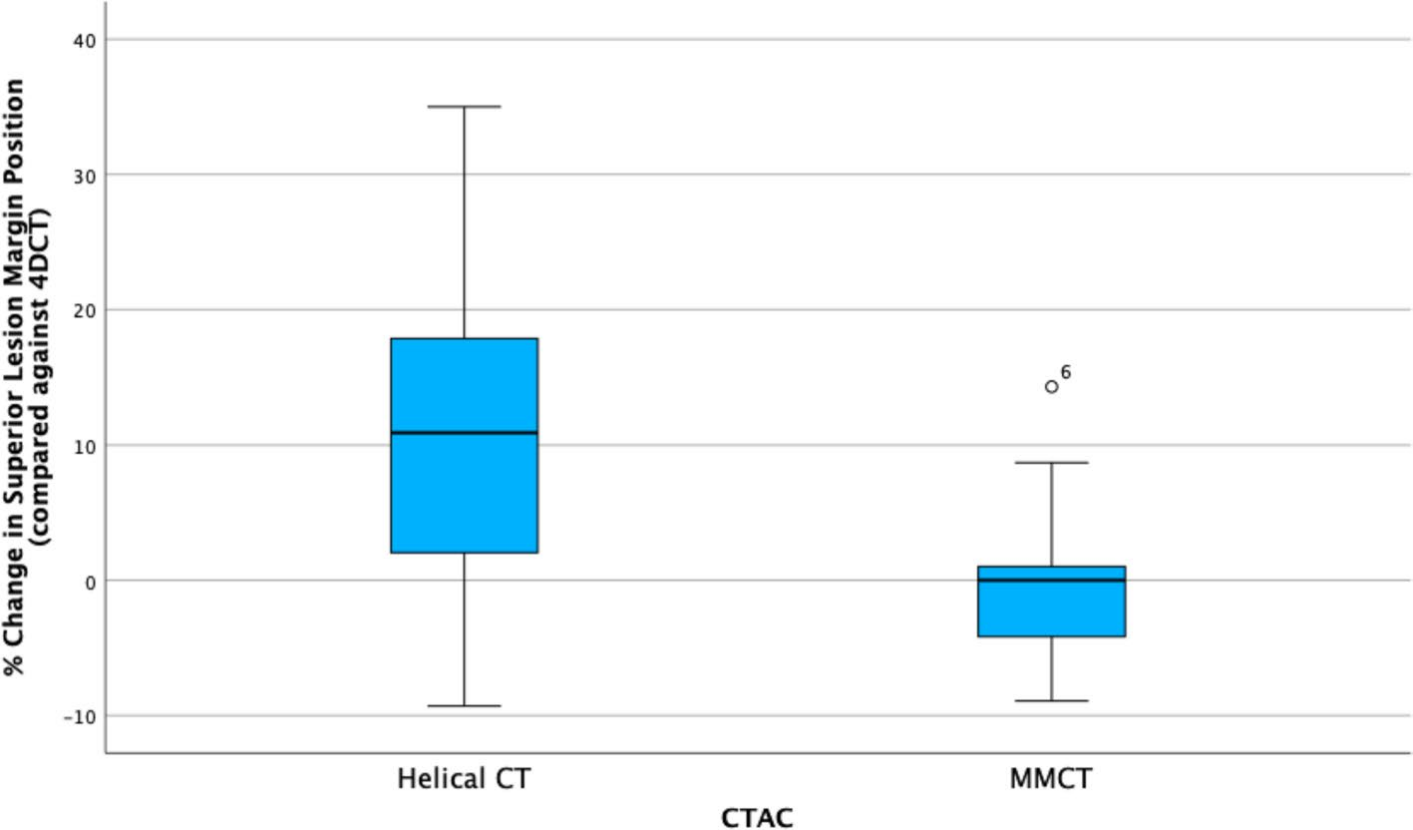
Boxplot illustrating differences in percentage change in superior lesion margin for $\hbox{CT}_{\text{helical}}$ and MMCT CTAC when compared against the 4DCT group

For the superior lesion margin, the repeated-measures one-way ANOVA suggests a statistically significant difference in position of the superior lesion margin within CTAC groups (Wilks’ $\lambda = 0.51$, $\hbox{F}(1.34, 21.38) = 12.67$, $p < 0.001$), which has been adjusted using Greenhouse-Geisser corrections for sphericty violation. The distributions of percentage change in superior lesion margin position are displayed in Fig. 6.

The mean percentage change in lesion position for the MMCT data is less than $\hbox{CT}_{\text{helical}}$ and considerably closer to zero, suggesting little percentage change compared to 4DCT, which indicates MMCT has an improved lesion margin position similarity than $\hbox{CT}_{\text{helical}}$. This can be confirmed by Bonferroni-corrected post hoc tests, which indicate a statistically significant difference between 4DCT and $\hbox{CT}_{\text{helical}}$ ($p = 0.007$) and no significant difference between 4DCT and MMCT ($p =1.0$). Consequently, it can be concluded that 4DCT and MMCT are equivalent for this measurement.

### Inferior lesion margin position

**Fig. 7:**
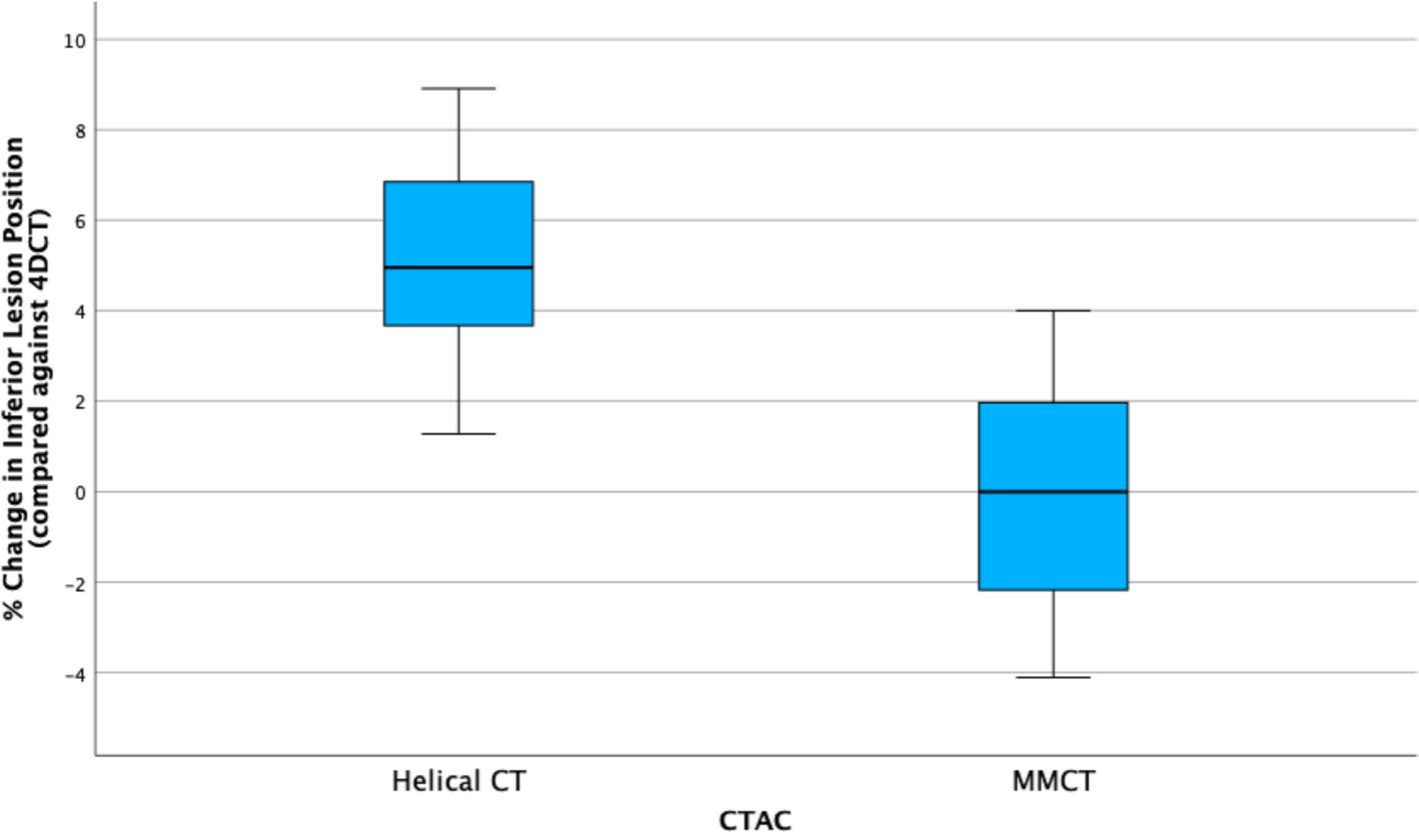
Boxplot illustrating differences in percentage change in inferior lesion margin for $\hbox{CT}_{\text{helical}}$ and MMCT CTAC when compared against the 4DCT group

Inferior lesion margin is used to denote the position of the bottom of the lesion. The repeated-measures one-way ANOVA indicates a statistically significant difference in inferior lesion margin position between CTAC groups (Wilks’ $\lambda = 0.18$, $\hbox{F}(2, 12) = 28.07$, $p <0.001$). The distributions of the percentage change in inferior lesion margin position are given in Fig. 7.

In this dataset, sphericity is assumed as per the Mauchly test, indicating that the variance within the populations are equal, so no correction is required. Post hoc tests revealed a significant difference between 4DCT and $\hbox{CT}_{\text{helical}}$ ($p < 0.001$) but no significant difference between 4DCT and MMCT ($p = 1.0$). Therefore, it can be deemed 4DCT and MMCT are equivalent concerning lesion positioning.

## Discussion

The capabilities provided by the MMCT algorithm include the ability to morph the widely available existing $\hbox{CT}_{\text{helical}}$ to best match the PET image, reducing PET/CT misregistration caused by patient respiratory motion. The MMCT can then mimic a 4DCT without the additional patient exposure or any specialist equipment or devices placed on the patient’s torso. The deviceless ability to retrospectively apply the MMCT algorithm infers reduced patient set-up time compared to external device use, and flexibility in image processing.

Results suggest no statistically significant differences in lesion position measurements between EE 4DCT and EE MMCT ($p = 1.0$). Consequently, lesion positions within CTs are equivalent in each case, and MMCT has been successfully warped to match the EE phase. From mean difference in position, the entire lesion position in the MMCT has a superior shift in comparison to $\hbox{CT}_{\text{helical}}$. Using MMCT, lesion positioning can be corrected without additional workflow demands and patient radiation dose arising from 4DCT acquisition. Liver measurements display a minimally significant difference between 4DCT and MMCT, potentially due to artefacts.

**Fig. 8:**
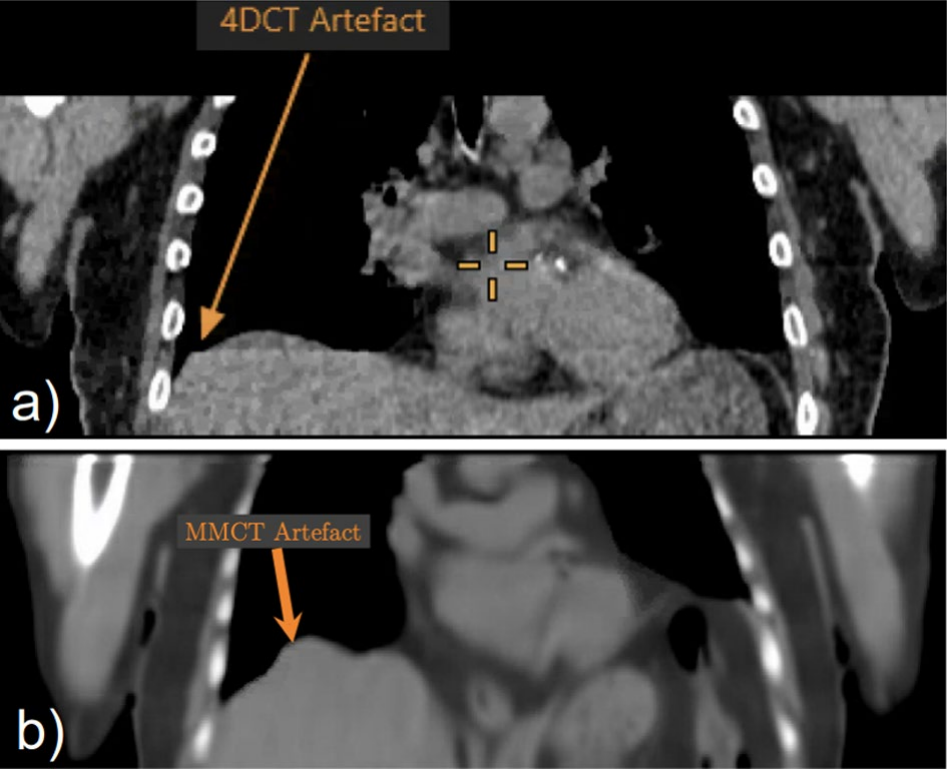
**a** 4DCT image illustrating artefact caused by the gating of the 4DCT file. **b** MMCT image illustrating the ’bubbled’ artefact across the liver margins

An artefact is present in some 4DCT images, likely due to being reconstructed in 4 cm blocks, which causes overlapping between each section. In addition, MMCT can have a “bubbled” liver artefact, displayed in Fig. 8, caused by noise within the dataset. This artefact may potentially impact lesion quantification for those close to the liver and will be investigated further in future work. Any methods that enable noise reduction within the dataset (for example, deep learning enhancement (DLE) [28]) could subsequently reduce this artefact.

Data from the inferior lesion margin position within CTAC groups indicates a greater change in mean difference compared to $\hbox{CT}_{\text{helical}}$, even when compared against the superior lesion. Decreased displacement of the inferior lesion margin indicates $\hbox{CT}_{\text{helical}}$ causes a stretching effect within the CT image. An interesting topic for further study would be to investigate non-linearity of motion within different lung regions [29]. Furthermore, the application of MMCT to dynamic data has not been examined in this work but would be a consideration for future research. Additional research is necessary to optimise the MMCT to the PET phase or to investigate the variation of the HU within the CT images.

Compared to previous research on PET/CT motion correction, our study highlights the potential influence of CT misregistration on PET imaging and offers a solution for phase-matched PET/CT without additional CT imaging using deformable registration algorithms. Warping the routinely acquired $\hbox{CT}_{\text{helical}}$ allows phase-matching to the PET image which would reduce the misregistration of the PET/CT and improve PET quantification. Therefore, this technique provides a low-dose alternative to the 4DCT acquisitions currently used for phase-matching.

In addition, the use of DDG-based methods, compared to an external device technique, provides many advantages. The DDG-based methods utilise a software-based approach compared to hardware, which is prone to signal or detection errors. This software approach also allows the motion correction to occur without any additional set-up time. The DDG-based methods extract motion from the activity within the images themselves compared to device-based methods which measure the amplitude of chest or abdominal wall motion, allowing for more robust measurement and motion detection. The ease of this device-less approach and reduced patient dose encourages routine implementation of DDG-based methods in PET/CT.

Drawbacks of this study include the limited dataset used to compare CTAC methods and that comparisons have been made for only the EE phase. Although all locally available exams were included, exams were excluded if they did not meet the threshold for gating in this study (R > 12) or due to a lack of data completeness- i.e. lack of completed scans or 4DCT gating errors. The EE phase was used for comparison as it is the phase generally used when correcting for respiratory motion when making a quiescent phase PET/CT image.

## Conclusion

The impact of motion in PET/CT imaging can give rise to motion artefacts and CTAC misregistration due to misalignments caused by respiratory motion during PET/CT acquisition. The MM algorithm creates a warped CT based on respiratory cycle phase estimation of images, reducing misregistration. The DDG-based MM method avoids any technical and exposure-related limitations of the 4DCT and improves workflow due to the absence of in-situ monitoring devices, making it more widely accessible.

This study demonstrates a clear proof-of-concept, utilising a patient dataset where acquired 4DCTs have been used to represent ground truth of motion quantification. CT analysis concludes an equivalency between MMCT and 4DCT at the EE phase, indicating that the algorithm can accurately map an arbitrary $\hbox{CT}_{\text{helical}}$ to a specified target phase, improving misregistration.

## Data Availability

The analysed data may be made available by the corresponding author upon reasonable request.

## Declarations

### Code availability

The DDG-based motion match algorithm code is copyrighted by GE HealthCare.
